# Ecthyma Gangrenosum-like Lesions in a Febrile Neutropenic Patient with Simultaneous Pseudomonas Sepsis and Disseminated Fusariosis

**DOI:** 10.4274/Tjh.2012.0030

**Published:** 2013-09-05

**Authors:** Seven Uludokumacı, İlker İnanç Balkan, Bilgül Mete, Reşat Özaras, Neşe Saltoğlu, Teoman Soysal

**Affiliations:** 1 İstanbul University Cerrahpasa Medical Faculty, Infectious Diseases and Clinical Microbiology İstanbul, Turkey; 2 İstanbul University Department of Infectious Diseases and Clinical Microbiology, Cerrahpaşa Faculty of Medicine İstanbul, Turkey

**Keywords:** Disseminated fusariosis, Pseudomonas aeruginosa sepsis, Ecthyma gangrenosum-like lesions

## Abstract

Fusarium spp. is an opportunistic mold that causes disseminated infections in immunocompromised patients. It is important to make a definite diagnosis because of high mortality rates. We present the case of a 27-year-old pregnant woman diagnosed with acute myeloid leukemia with a prolonged febrile neutropenic period. She developed ecthyma gangrenosum-like lesions and simultaneously had Pseudomonas bacteremia and disseminated fusariosis. Histopathological and microbiological features of skin lesions had a critical role in differential diagnosis. Ecthyma gangrenosum-like lesions due to disseminated fusariosis might be easily misdiagnosed as lesions associated with Pseudomonas unless tissue cultures and histopathological examinations are performed.

**Conflict of interest:**None declared.

## Abstract (tr)

Fusarium spp. immündüşkün hastalarda dissemine enfeksiyonlara neden olan oportünistik bir küf mantarıdır. Yüksek mortalite oranları nedeniyle kesin tanıya gitmek önemlidir. Sunmakta olduğumuz olguda uzamış febril nötropenik dönemde bulunan AML tanılı 27 yaşında bir gebede Pseudomonas bakteremisi ile dissemine fusariozis eşzamanlı gelişmiş ve aynı dönemde ektima gangrenozum benzeri lezyonlar ortaya çıkmıştır. Ayırıcı tanıda deri lezyonlarının histopatolojik ve mikrobiyolojik özellikleri önemli bir yer tutmuştur. Dissemine fusariozise bağlı ektima gangrenozum benzeri lezyonlar doku kültürü ve histopatolojik incelemeler yapılmazsa kolaylıkla Pseudomonas enfeksiyonu ile ilişkili lezyonlarla karıştırılabilir.

## INTRODUCTION

Fusarium spp. is an opportunistic mold that causes disseminated infections in immunocompromised patients [1]. Cutaneous lesions like ecthyma gangrenosum are seen in 70% of the cases [2,3]. We present a patient with acute myeloid leukemia in a febrile neutropenic period with ecthyma gangrenosum-like cutaneous lesions who had Pseudomonas bacteremia and disseminated fusariosis simultaneously. An Internet search of the English-language literature (Medline 1966-2011) revealed no reported cases of ecthyma gangrenosum-like lesions due to simultaneous Pseudomonas bacteremia and disseminated fusariosis. We aim to draw attention to the cutaneous lesions in immunocompromised patients, as they may cause diagnostic challenges because of being associated with different invasive etiologic agents while having similar appearances. 

## CASE REPORT

A 27-year-old patient, 9 weeks pregnant and diagnosed with acute myeloid leukemia, was admitted with a febrile neutropenic attack and initially treated empirically with ceftazidime. On the fifth day of admission, conventional amphotericin B (Amp B) at 1.2 mg/kg/day was added to the treatment because of persistent fever. Computed tomography (CT) of the thorax revealed multiple nodules on the right lung. On day 8, medical abortion was performed. After abortion, her general condition worsened, respiratory distress developed, and the treatment was switched to meropenem (1 g, q8h) and levofloxacin (750 mg, q24h). The patient was transferred to the intensive care unit (ICU) and underwent chemotherapy (ARA-C, 170 mg/day D1-7). After 2 weeks of follow-up in the ICU, her general condition was partially improved and she was taken back to the hematology department. In the first month of antifungal therapy, follow-up thoracic CT demonstrated progression of acinar nodules and consolidations bilaterally in the lung parenchyma. Serum galactomannan test results were found positive 2 times consecutively. With preliminary diagnosis of probable invasive pulmonary aspergillosis, Amp B was switched to voriconazole (loading dose of 6 mg/kg, q12h, 2 doses; maintenance of 4 mg/kg, q12h).

After one month of this therapy, because of persisting fever, bronchoscopy was performed. In the bronchoalveolar lavage culture, Chryseobacterium indologenes and Candida glabrata (voriconazole MIC: 4 µg/mL) were isolated. Although colonization of these microorganisms could not be excluded, because of the persisting fever and high levels of acute phase reactants, these agents were accepted as pathogenic microorganisms. Treatment was changed to caspofungin (50 mg/day) and piperacillin/tazobactam (PIP/TAZ; 3 × 4.5 g/day) according to the susceptibility tests. 

**Figure 1:**
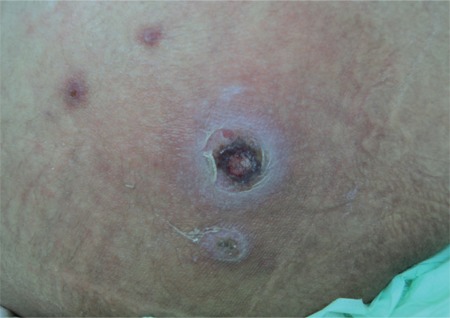
Ectyhma gangrenosum related to pseudomonas septicemia (center) and ecthyma gangrenosum-like lesions related to fusariosis (upper left corner).

**Figure 2:**
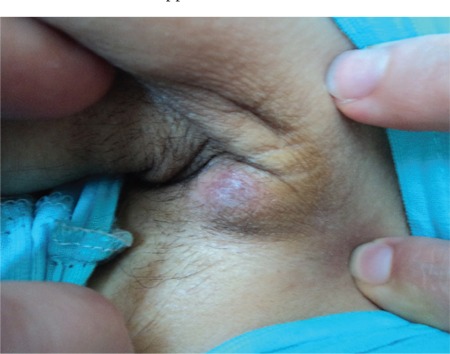
Axillary nodular skin lesion related with fusariosis

**Figure 3:**
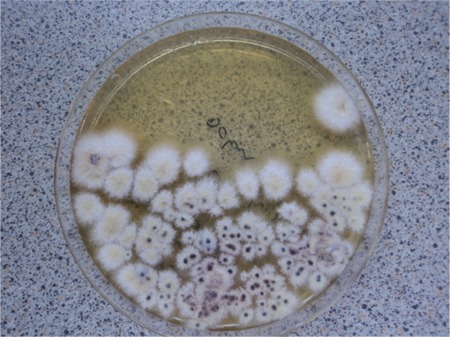
Fusarium spp. colonies grown from skin biopsy specimen culture

**Figure 4:**
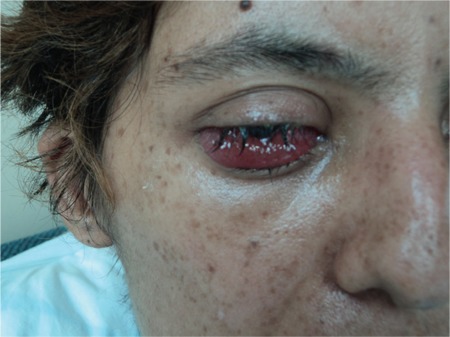
Eye involvement of disseminated fusariosis

During the patient’s follow-up, clinical signs due to the progression of leukemia were detected. On days 83-88 of admission, fludarabine + ARA-C chemotherapy was given. On day 17 of PIP/TAZ therapy, while fever and neutropenia were still persisting, an ulcerated erythematous lesion with a necrotic center was detected on the patient’s right hip, and there were smaller acneiform, erythematous nodular lesions on the left side of the abdominal wall, the left hip, the forearm, and the axilla (Figures 1 and 2). During the infection period, a total of 8 sets of hemocultures were obtained. Pseudomonas aeruginosa was isolated in 4 sets of hemocultures. Ciprofloxacin (2×400 mg/day) and amikacin (1×1 g/day) were added to PIP/TAZ according to the susceptibility tests. Histopathological analysis of the skin lesion on the right hip revealed ecthyma gangrenosum. Fusarium spp. was isolated from the skin biopsy of the erythematous nodular lesions on the left hip (Figure 3). Simultaneously, the patient complained of loss of vision in her right eye, and fundoscopic examination revealed fungal endophthalmitis (Figure 4). Liposomal Amp B was added to caspofungin, but because of severe allergic reaction, it was replaced by voriconazole. Informed consent was obtained.

Combined antibacterial therapy for Pseudomonas bacteremia was completed in 3 weeks. With daily granulocyte colony stimulating factor therapy, the patient became non-neutropenic and her follow-up bone marrow biopsy revealed signs of remission. A convulsion occurred on day 21 of combined antifungal therapy. Cranial magnetic resonance imaging showed disseminated lesions compatible with fungal infection. Antifungal treatment was continued and remission of lesions was observed 2 weeks later. After combination therapy for one month, she was discharged from the hospital with oral voriconazole. During one year of follow-up after discharge, the patient remained in remission hematologically and voriconazole was ceased at month 9 with resolution of radiological signs due to pulmonary fungal infection.

## DISCUSSION

Co-infections of Fusarium spp. and P. aeruginosa are rarely reported [4]. Fusarium spp. is a mold that may cause superficial infections like keratitis and onychomycosis in immunocompetents, as well as severe disseminated or locally invasive (lung, sinuses, etc.) infections in immunocompromised patients [3]. In patients with hematological malignancies and prolonged fever and neutropenia, it is important to make a differential diagnosis between fusariosis and invasive pulmonary aspergillosis because of their similar clinical presentations. Negativity of galactomannan antigen and absence of typical radiological signs of aspergillosis can favor a diagnosis of fusariosis, whereas recent studies reported that galactomannan can also be positive in cases of fusariosis [5,6]. Disseminated fusariosis is described as the involvement of two or more non-contiguous regions [7]. Skin involvement is the first clue in most disseminated fusariosis cases and often occurs at an early stage of the disease [8]. Multiple erythematous macular or papular painful lesions are reported in 70% of cases. Lesions usually have a necrotic center resembling ecthyma gangrenosum and are described as ecthyma gangrenosum-like lesions [3]. In our case, there were two different types of cutaneous involvement. One of them was consistent with fusariosis, whereas other lesions were evaluated as ecthyma gangrenosum secondary to P. aeruginosa bacteremia. To our knowledge, this is the first case reported in the literature of ecthyma gangrenosum-like lesions with simultaneous Pseudomonas sepsis and disseminated fusariosis. In the management of fusariosis, conventional or liposomal Amp B may be used singly; successful results were achieved also by combining it with voriconazole or posaconazole [8,9,10]. In the present case, voriconazole was combined with caspofungin instead of liposomal Amp B and complete response to treatment was achieved, with total resolution of cranial and pulmonary lesions.

## CONCLUSION

It is crucial to carefully detect every single skin lesion in patients with hematological malignancies. These lesions could be incorrectly attributed to Pseudomonas aeruginosa bacteremia and disseminated fusariosis would easily be overlooked unless tissue cultures and histopathology were performed.

## CONFLICT OF INTEREST STATEMENT

The authors of this paper have no conflicts of interest, including specific financial interests, relationships, and/ or affiliations relevant to the subject matter or materials included.
